# Investigation on Physicochemical Characteristics of a Nanoliposome-Based System for Dual Drug Delivery

**DOI:** 10.1186/s11671-018-2519-0

**Published:** 2018-04-13

**Authors:** Jae Hyun Nam, So-Yeon Kim, Hasoo Seong

**Affiliations:** Therapeutics and Biotechnology Division, Korea Research Institute of Chemical Technology, 141 Gajeong-ro, Yuseong-Gu, Deajeon, 34114 Republic of Korea

**Keywords:** Nanoliposome, Dual drug delivery system, Ultrasonication, Remote loading technology, Time-differential release

## Abstract

Synergistic effects of multiple drugs with different modes of action are utilized for combinatorial chemotherapy of intractable cancers. Translation of in vitro synergistic effects into the clinic can be realized using an efficient delivery system of the drugs. Despite a few studies on nano-sized liposomes containing erlotinib (ERL) and doxorubicin (DOX) in a single liposome vesicle, reliable and reproducible preparation methods as well as physicochemical characteristics of a non-PEGylated nanoliposome co-encapsulated with ERL and DOX have not been yet elucidated. In this study, ERL-encapsulated nanoliposomes were prepared using the lipid film-hydration method. By ultrasonication using a probe sonicator, the liposome diameter was reduced to less than 200 nm. DOX was loaded into the ERL-encapsulated nanoliposomes using ammonium sulfate (AS)-gradient or pH-gradient method. Effects of DOX-loading conditions on encapsulation efficiency (EE) of the DOX were investigated to determine an efficient drug-loading method. In the EE of DOX, AS-gradient method was more effective than pH gradient. The dual drug-encapsulated nanoliposomes had more than 90% EE of DOX and 30% EE of ERL, respectively. Transmission electron microscopy and selected area electron diffraction analyses of the dual drug-encapsulated nanoliposomes verified the highly oriented DOX-sulfate crystals inside the liposome as well as the less oriented small crystals of ERL in the outermost region of the nanoliposome. The nanoliposomes were stable at different temperatures without an increase of the nanoliposome diameter. The dual drug-encapsulated nanoliposomes showed a time-differential release of ERL and DOX, implying proper sequential releases for their synergism. The preparation methods and the physicochemical characteristics of the dual drug delivery system contribute to the development of the optimal process and more advanced systems for translational researches.

## Background

Intractable cancers such as triple-negative breast cancers have limits to treatment with standard therapies because of DNA damage response highly interconnected with various signaling networks [1–3]. Therapeutic strategies increasing initial chemo-sensitivity of such recalcitrant tumors have been studied to challenge the limits [4, 5]. A recent study suppressing oncogenic signaling pathways while using a DNA damage agent is one of the combinatorial therapeutic strategies [6–8]. The study suggested that pretreatment of a growth factor inhibitor to resistant cancer cells synergize their apoptotic response to a genotoxic drug [9, 10]. There is a highly important clinical need for therapeutic strategies to target multiple cancer cell-specific survival pathways for enhancement of the extent of tumor cell killing and potential reduction of total drug exposure during treatment [11, 12]. Meanwhile, in order to translate in vitro synergistic effects of two kinds of drugs into the clinic, a delivery system that can deliver the both drugs and release them in time-differential manner is essential due to the difference between pharmacokinetic (PK) properties of the drugs and the difficulty in targeting the same cancer cells in proper temporal sequence [13–16].

A nanoliposomal delivery system is one of the dual drug delivery systems which can be applied to combination therapy. The major components of liposomes are in general phospholipids similar to those of cell membranes. The phospholipids form a bilayered concentric sphere with an inner compartment and bilayer membranes [17]. The nanoliposomes can allow the drugs having different physicochemical properties to be loaded into the inner compartment and the bilayer membrane of the liposomes and then delivered to the lesion. Thus, in vivo synergistic effect of the two drugs delivered using the nanoliposomal system can be achieved through enhanced co-localization of the drugs to cancer cells by not only reconciliation of the PK properties of each drug but also the so called enhanced permeability and retention (EPR) effect of tumor tissue. Besides the encapsulation of drugs in the inner compartment, the nanoliposome can solubilize hydrophobic drugs, enhance their stability, modulate their blood circulation properties, and hence induce their accumulation to tumor tissues [18].

Despite the recent studies on nanoliposome-based combination chemotherapy delivery systems, the preparation processes of the systems need to be optimized for reliable and reproducible fabrication, and physicochemical characteristics of them should be elucidated for the development of more advanced systems [13, 19]. In our study, a nanoliposomal delivery system co-encapsulated with elrotinib (ERL; ERL free base, log_P_ = 3.3) and doxorubicin (DOX; DOX HCl, log_P_ = 1.27) in a single liposome vesicle was prepared as a model nanoliposome system according to a previous report except for a so-called non-PEGylated liposome formulation [13], and physicochemical characteristics of the system were studied. In vitro drug release studies were carried out to investigate a time-differential release of the drugs. Among several methods such as extrusion, sonication, and high-pressure homogenization [20–22], ultrasonication using a probe sonicator was utilized to reduce liposome diameter due to its more efficient processability. A comparative study about effects of drug-loading technologies such as pH- or ammonium sulfate-gradient method on encapsulation efficiency (EE) of the drug was performed to optimize the drug encapsulation. In addition, the most effective process for drug encapsulation was determined through investigating the effects of process conditions on EE of the drug. The preparation conditions for nanoliposomal dual drug delivery system encapsulated with both ERL and DOX and releasing the drugs in a proper sequence were optimized. The optimized preparation methods and the characteristics of the dual drug delivery system suggest a platform of a reliable and reproducible preparation processes and a more advanced delivery system for translational studies.

## Methods

### Materials

1,2-Distearoyl-*sn*-glycero-3-phosphocholine (DSPC) and 1-palmitoyl-2-oleoyl-*sn*-glycero-3-phospho-(1′-*rac*-glycerol) (sodium salt) (POPG) were supplied from Avanti Polar Lipids, Inc. (Alabaster, Al, USA). Cholesterol was from Sigma-Aldrich, Corp. (St. Louis, MO, USA). Doxorubicin (DOX) hydrochloride and erlotinib (ERL) free base were purchased from Boryung Co., Ltd. (Seoul, Korea) and Shanghai Send Pharmaceutical Technology Co., Ltd. (Shanghai, China), respectively. Ammonium sulfate (AS) and citric acid (CA) anhydrous were supplied from Daejung Chemicals & Metals Co., Ltd. (Siheung-si, Korea). All the other reagents were of reagent grade and supplied from Sigma-Aldrich, Corp. (St. Louis, MO, USA).

### Preparation of Dual Drug-Encapsulated Nanoliposomes

**Fig. 1:**
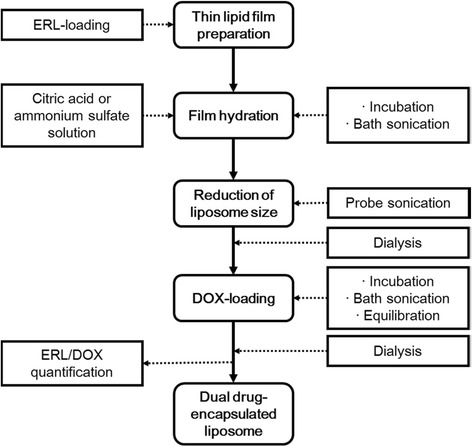
Processes for preparation and characterization of dual drug-encapsulated nanoliposomes

General processes for preparing the nanoliposomes encapsulated with both ERL and DOX are depicted in Fig. 1. In brief, ERL-encapsulated liposomes were prepared using the so-called thin lipid film-hydration method [23]. ERL was added to the lipid mixture forming the thin lipid film in order to encapsulate the drug into lipid bilayer membrane of the liposomes. The liposome diameter was reduced by ultrasonication with a probe sonicator (Vibra Cell; Sonics & Materials, Inc., Newtown, CT, USA). DOX was encapsulated into the inner aqueous compartment of ERL-encapsulated nanoliposomes using a transmembrane ion concentration gradient of the nanoliposomes. The detailed experimental and analytical methods of the process are suggested in the following sections.

#### ERL-Encapsulated Liposomes

A lipid mixture composed of DSPC, cholesterol, and POPG at a ratio of 27:20:3 (*w*/*w*/*w*) was mixed with ERL free base at a weight ratio of the drug to the total lipid, 3:50. Those mixtures were dissolved in 9 mL of a mixed solvent of chloroform:methanol = 2:1 (*v*/*v*). The lipid solution was placed in a round-bottomed flask, and to form a lipid membrane, the solvent was removed using a rotary evaporator (Rotavapor R-210; BÜCHI Labortechnik AG, Flawil, Switzerland) under vacuum at 40 °C. The membrane was dried in a vacuum overnight to remove all the residual solvent. In these processes, ERL molecules are inserted into the lipid membrane via hydrophobic attractions and then spontaneously encapsulated into the lipid bilayer membrane of the liposomes, which are formed by the following hydration process.

In order to hydrate the ERL-containing lipid membrane, 8 mL of 250–400 mM AS or 300 mM citric acid buffer (pH 3.9) were added to the membrane formed at the inner surface of round-bottomed flask. After incubation of the membrane at 65 °C for 30 min by rotation of the flask, the liposome suspension was sonicated using a bath sonicator (Branson 3510E-DTH; Branson, Danbury, CT, USA). To reduce the liposome diameter, the liposome suspension was sonicated using the probe sonicator at the conditions such as a pulse of 5 s on and 2 s off, an amplitude of 20% and an energy of 36 J per pulse in an ice-water bath, or a water bath under magnetic stirring. To remove AS which was unloaded into the nanoliposomes, dialysis of the nanoliposomes was performed overnight in phosphate buffered saline (PBS, pH 7.4) with a dialysis tubing cellulose membrane (14,000 molecular weight cutoff (MWCO); Sigma-Aldrich Corp., St. Louis, MO, USA). In the case of nanoliposome suspension prepared using the citric acid buffer, the pH of the solution was adjusted to about 6.5 using 300 mM sodium bicarbonate buffer to make a gradient between the inside and outside of the nanoliposomes.

#### DOX-Loading into ERL-Encapsulated Nanoliposomes

To encapsulate DOX into ERL-encapsulated nanoliposomes, DOX hydrochloride was first dissolved in 0.9% NaCl aqueous solution at 65 °C. In general, 2 mL of 1.5 mg/mL DOX solution were added to 8 mL of the AS- and ERL-encapsulated or citric acid- and ERL-encapsulated nanoliposome suspension. Drug-loading processes of the DOX-added nanoliposome suspension were composed of incubation, sonication using the bath sonicator, equilibration at room temperature (RT), and dialysis against PBS (pH 7.4) using the dialysis tubing cellulose membrane to remove unencapsulated DOX from the nanoliposome suspension. Effects of DOX-loading conditions on DOX’s EE were investigated using four experimental groups. Group 1 was the mixture of DOX solution and the liposomes, which was treated in the sequence of an incubation at 65 °C for 30 min, a sonication at 65 °C for 5 min, and an overnight dialysis. Group 2 was incubated at 65 °C for 30 min, sonicated at 65 °C for 5 min, equilibrated for 30 min at RT, and then dialyzed overnight. Group 3 was incubated at 65 °C for 30 min, sonicated at 65 °C for 15 min, and then dialyzed overnight. Group 4 was incubated at 65 °C for 30 min, sonicated at 65 °C for 15 min, equilibrated for 30 min at RT, and then dialyzed overnight.

### Diameter, Morphology, and Physical Stability of Dual Drug-Encapsulated Nanoliposomes

Diameter of the drug-encapsulated nanoliposomes was measured at 25 °C using a particle size analyzer (ELS-Z; Otsuka Electonics Co., Ltd., Osaka, Japan). Morphology and mean diameter of the single or dual drug-encapsulated nanoliposomes were observed using a field emission transmission electron microscope (FE-TEM) (JEM 2100F; JEOL Ltd., Tokyo, Japan, installed at Korea Basic Science Institute) at 200 kV. In order to prepare the sample, the nanoliposome suspension was drop-casted onto a carbon-coated copper grid, and the grid was air-dried at RT before viewing under the microscope. Physical stability of the dual drug-encapsulated nanoliposomes incubated in PBS (pH 7.4) at different temperatures of 4, 25, and 37 °C was investigated by monitoring a change in the liposome diameter as a function of time. The liposome diameter was measured using a particle size analyzer (Zetasizer Nano ZS, Malvern Instruments Limited, Worcestershire, UK).

### EE of Drugs

Encapsulation efficiency (EE) of DOX or ERL in the dual drug-encapsulated nanoliposomes was determined through the following Eq. (1).

1 $$ \mathrm{EE}\ \left(\%\right)={C}_f/{C}_i\times 100 $$

where *C*_*f*_ is the encapsulated amount of ERL or DOX in the nanoliposomes measured after destruction of the nanoliposomes with 10% Triton-X 100 for a complete release of ERL or DOX from them. The absorbance of ERL was measured using a UV-Vis spectrometer (DU-800 spectrophotometer; Backman Coulter Inc., Fullerton, CA, USA) at 345 nm and fluorescence intensity of DOX was using a spectrofluorometer (SFM-25; Tegimenta AG, Rotkreuz, Switzerland) at 495 and 590 nm of excitation and emission wavelengths, respectively. The amount of ERL or DOX was calculated using calibration curve of ERL or DOX prepared in advance. *C*_*i*_ is the ERL or DOX amount added to the lipid mixture or ERL-encapsulated nanoliposomes. The amounts were determined measuring the absorbance or fluorescence intensity of each drug at corresponding wavelengths.

### Drug Release

A dialysis cassette (10,000 MWCO, Slide-A-Lyser Dialysis Cassette G2; Thermo Scientific Corp., Rockford, IL, USA) was filled with the suspension of nanoliposomes encapsulated with both DOX and ERL. The cassette was placed in PBS (pH 7.4), and the release test media were stirred continuously at 37 °C. At predetermined time points, aliquots of the sample were taken from the cassette to determine the concentration of each drug and then the drug release was calculated through the following Eq. (2).

2 $$ \mathrm{Drug}\ \mathrm{release}\ \left(\%\right)=\left({D}_i-{D}_t\right)/{D}_i\times 100 $$

where *D*_*t*_ is the concentration of DOX or ERL remaining in the nanoliposomes at a given time during the release test period and *D*_*i*_ is the concentration of DOX or ERL encapsulated in the nanoliposomes before the drug release experiment. The DOX concentration was determined measuring fluorescence intensity of the sample with the fluorescence spectrometer at 495 and 590 nm of excitation and emission wavelengths, respectively. The ERL concentration was determined measuring absorbance of the sample at 345 nm with the UV-Visible spectrometer.

### Statistical Analysis

Results are presented as mean ± SEM, unless otherwise noted.

## Results and Discussion

### Effect of Preparation Processes on Nanoliposome Characteristics

**Fig. 2:**
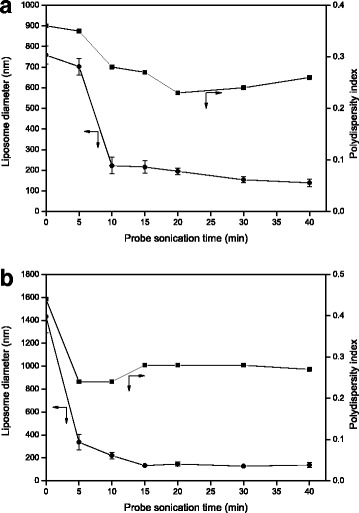
Effects of probe sonication time on diameter and polydispersity index of ERL-encapsulated nanoliposomes: an ice-water bath (**a**) or a water bath (**b**) cooling of liposome suspension in a container during ultrasonication (*n* = 3, the data are presented as mean ± SEM)

Dual drug-encapsulated nanoliposomes were prepared encapsulating ERL into lipid bilayer membrane and DOX into inner compartment of the nanoliposomes. Liposomes encapsulated with ERL only were prepared hydrating ERL-incorporated lipid membrane with an aqueous AS solution. During these processes, ERL molecules are incorporated into lipid membrane via hydrophobic attractions and then spontaneously encapsulated into lipid bilayer membrane of the liposomes. After the hydration, the liposome suspension was ultrasonicated using the horn-type probe sonicator to reduce the liposome diameter. To investigate effects of the ultrasonication and the cooling methods on the liposome diameter, the liposomes were treated with an increase of sonication time under different cooling conditions and the results are shown in Fig. 2. Figure 2a represents the effect of the ultrasonication time on the diameter and polydispersity index (PDI) of the liposomes treated under the cooling condition of an ice-water bath. The diameter of ERL-encapsulated liposomes untreated by the ultrasonication was 759 ± 44 nm. However, until 10 min of ultrasonication, the liposome diameter decreased remarkably to 222 ± 40 nm, and after 10 min, there was no significant change of the diameter as the ultrasonication time increased. These results indicate that ERL-encapsulated liposomes formed by the hydration are mostly multilamellar vesicles (MLVs). The high energy delivered to MLV-type liposomes via the ultrasonication exfoliates the multilayer of MLVs and disassembles the large liposomes. In aqueous solution, the lipids separated from the MLVs are self-assembled through hydrophobic attractions and thus transformed into large unilamellar vesicle (LUV)––or small UV (SUV)-type nanoliposomes. As these phenomena repeat, the mean nanoliposome diameter gradually decreases [24–26]. As shown in Fig. 2a, the diameter of the liposomes was remarkably reduced in a short period of ultrasonication. It is thought that during this period, most of the MLV-type liposomes were converted to LUV- or SUV-type liposomes [27]. The ultrasonication longer than 10 min caused a slight decrease of the liposome diameter, indicating less transformation of the vesicle type with the ultrasonication time. The PDI of the liposome diameter decreased with an increase of the ultrasonication time, suggesting that the liposome diameter became uniform with the time in spite of a slight increase of the PDI after 20 min. Figure 2b represents the effect of ultrasonication time on the diameter and PDI of the liposomes treated under cooling condition of a water bath. The diameter of the liposomes untreated by the ultrasonication was 1433 ± 143 nm. The liposome diameter decreased significantly to 337 ± 67 nm until 5 min of ultrasonication, and after 5 min, there was a gradual decrease of the diameter until 15 min, followed by little change in the diameter. The PDI of the liposome diameter varied with the ultrasonication time until 15 min and reached a plateau thereafter. Under the water bath condition, the time spent for reducing the liposome diameter to below 30% of the diameter of untreated liposomes was shorter than the time spent under the ice-water bath condition. It is thought that the reduction of the liposome diameter was made by transfer of the thermal energy generated by the ultrasonication to MLV-type liposomes. Compared to the ice-water bath condition, the temperature of the liposome suspension sonicated under the water bath condition was raised too high, implying that the heat of the liposome suspension which took place due to a bulk heating by ultrasonic cavitation was less efficiently released. To keep the liposome suspension from side effects such as a medium evaporation and a possible deterioration of lipid molecules induced by overheating, the ice-water bath was chosen as the cooling condition for the ultrasonication. On the other hand, the ultrasonicated liposomes were dialyzed to remove unencapsulated AS. The ERL- and AS-encapsulated nanoliposomes prepared using the ultrasonication and the dialysis had about 140 nm of the mean nanoliposome diameter, suggesting a slight decrease of the nanoliposome diameter through the dialysis. The ERL- and AS-encapsulated nanoliposomes were utilized for DOX encapsulation into inner compartment of the nanoliposomes using AS-gradient method.

**Fig. 3:**
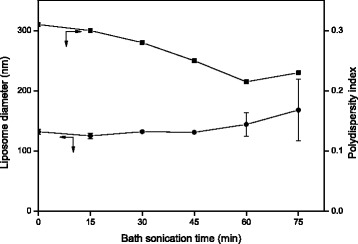
Effects of bath sonication time on diameter and polydispersity index of DOX- and ERL-encapsulated nanoliposomes (*n* = 3, the data are presented as mean ± SEM)

Figure 3 shows the diameter of DOX- and ERL-encapsulated nanoliposomes according to the time of ultrasonication using a bath sonicator during a DOX-loading process. Low variations of the diameter were similar to those after 10 min of ultrasonication of ERL-encapsulated nanoliposomes as shown in Fig. 2a. In spite of the increase of ultrasonication time, the nanoliposome diameter did not change significantly until 45 min of ultrasonication. These results can be attributable to the fact that DOX- and ERL-encapsulated nanoliposomes are LUVs or SUVs [27]. Meanwhile, when the ultrasonication time was longer than 45 min, the increase of the SEM of mean nanoliposome diameter occurred, indicating an increase of differences among the liposome samples. The PDI of the diameter decreased as the ultrasonication time increased. These results suggest that the uniformity of the liposome diameter increased with the ultrasonication time in spite of a slight increase of the PDI after 60 min of ultrasonication. In short, the change in nanoliposome diameter by ultrasonication was the highest in the transformation stage where the liposomes would be converted from MLVs to LUVs or SUVs. Therefore, the ultrasonication for reduction of the liposome diameter was the most effective when the treatment was performed between the hydration of lipid membrane and the DOX encapsulation into inner compartment of ERL-encapsulated nanoliposomes for a relatively short duration.

### Effect of Drug-Loading Method on EE

**Fig. 4:**
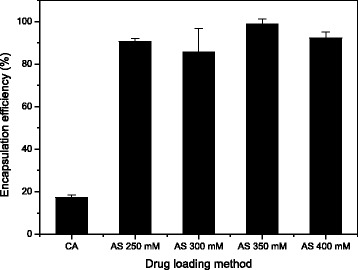
Encapsulation efficiency of DOX in dual drug-encapsulated nanoliposomes according to DOX-loading methods and ammonium sulfate (AS) concentrations: CA citric acid (*n* = 3, the data are presented as mean ± SEM)

The dual drug-encapsulated nanoliposomes were prepared using DOX-loading into ERL-encapsulated nanoliposomes with approximately 30% EE of ERL and 140 nm of the nanoliposome diameter. The amount of encapsulated DOX or ERL in the liposomes was measured after destruction of the liposomes with a surfactant for complete releases of the drugs from the liposomes. The fluorescence intensity of DOX and the absorbance of ERL were measured by spectrofluorometry and UV-Vis spectrometry, respectively. To investigate effects of DOX-loading methods on EE of DOX in ERL-and DOX-encapsulated nanoliposomes, active loading method such as pH-gradient or AS-gradient method was utilized for DOX encapsulation into the liposomes. The difference in EE between the two drug-loading methods was investigated, and the results are shown in Fig. 4. The EEs of DOX by using AS-gradient method were 90% or more, and the EE by using pH-gradient was about 17%, indicating that AS-gradient method was far more effective in DOX encapsulation than pH-gradient method [28].

When the changes in the EEs of DOX according to the AS concentrations were compared, the EE was the highest and SEM of the EE was the lowest at 350 mM AS. AS-gradient or pH-gradient method utilizes a drug translocation mechanism, which induces the drugs outside the nanoliposomes to migrate into inner compartment of the nanoliposomes because of a driving force generated from a transmembrane ion concentration gradient of the nanoliposomes [29, 30]. The EE of DOX by AS-gradient method was, however, quite higher than that of pH-gradient method. These results can be ascribed to the fact that AS-gradient method is more effective in developing crystalline complexes than pH-gradient method due to higher crystallizability between the ionized DOX and the counter ions inside the nanoliposomes [31, 32].

### Effect of Drug-Loading Conditions on EE

**Fig. 5:**
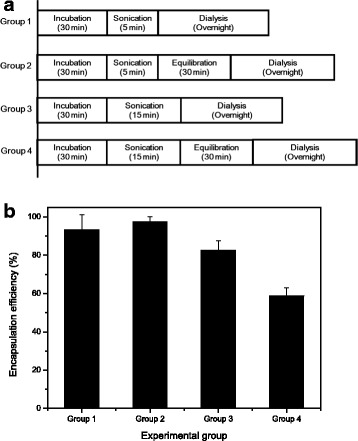
Encapsulation efficiency of DOX in dual drug-encapsulated nanoliposomes according to various drug-loading conditions: drug-loading conditions for each experimental group (**a**) and drug encapsulation efficiencies of the experimental groups (**b**) (*n* = 3, the data are presented as mean ± SEM)

To investigate the effect of treatment conditions for a mixture of DOX solution and ERL-encapsulated nanoliposomes on DOX’s EE during the DOX-loading process, four experimental groups shown in Fig. 5a were designed and experimented according to the different treatment conditions. Group 1 was the mixture treated in the sequence of an incubation, a sonication, and an overnight dialysis. In group 2, to investigate effect of an equilibration of the sonicated mixture on the EE, the mixture was incubated, sonicated, equilibrated for 30 min at RT, and then dialyzed overnight. As a group for investigating effect of a long-term sonication on the EE, group 3 was the mixture incubated, sonicated at 65 °C for 15 min, and then dialyzed overnight. Moreover, as a group for investigating effects of a long-term sonication and an equilibration on the EE, group 4 was the mixture incubated, sonicated at 65 °C for 15 min, equilibrated for 30 min at RT, and then dialyzed overnight. In all groups, DOX-loading was carried out using AS-gradient method. As shown in Fig. 5b, the EEs of DOX of the groups 1, 2, 3, and 4 were 93 ± 7.8, 98 ± 2.5, 83 ± 4.9, and 59 ± 4.2%, respectively. In comparison to the EE of group 3, the EE of group 1 was higher, indicating that a shorter duration of bath sonication is more effective treatment to increase the EE in the nanoliposomes. When compared with the EE of group 1, group 2 had higher EE of DOX. The EE of group 4 was the lowest among all the groups experimented. In contrast to the EE enhancement by the equilibration after a short-term sonication applied to group 2, the equilibration after a long-term sonication applied to group 4 was not efficacious for the EE enhancement. These results may be due to the excessive amount of energy delivered to the suspension, which could disrupt the nanoliposomes during the bath sonication described above. Also, it is thought that by long-term bath sonication in groups 3 and 4, the EE was lowered because of a decrease of AS content in the nanoliposomes. Since the temperature of the nanoliposome suspension increased far beyond the phase-transition temperature (*T*_*c*_) by the long-term bath sonication, the fluidity of the lipid bilayer increased and therefore induced the AS release from the liposomes. The nanoliposome samples of group 3 were dialyzed immediately after the bath sonication, suggesting a rapid cooling of the samples. In contrast, the nanoliposome samples of group 4 were treated by the long-term bath sonication followed by the equilibration, which might sustain the AS release, increase the formation of DOX-sulfate complex outside the liposomes, and therefore lower the EE. Although the equilibration after the long-term sonication was not effective, these results suggest that the DOX-loading process such as a sequential treatment of mixture of the DOX solution and the ERL-encapsulated nanoliposomes–an incubation above *T*_*c*_ of phospholipid, a bath sonication, an equilibration below the *T*_*c*_, for example, at RT, and an overnight dialysis–is a more effective method to increase the EE than other processes without the equilibration treatment [33–35].

### Morphology and Physical Stability of Dual Drug-Encapsulated Nanoliposomes

**Fig. 6:**
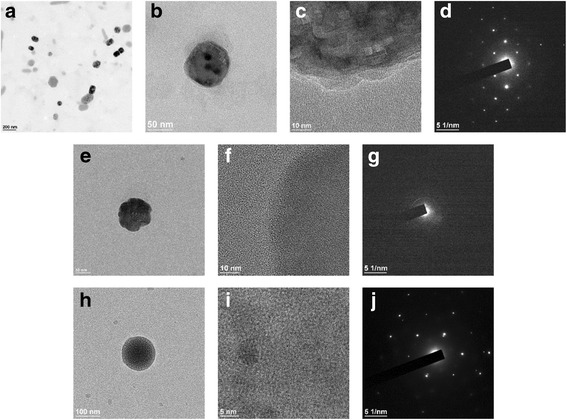
Transmission electron microscopy (TEM) and selected area electron diffraction (SAED) analyses of dual drug- or single drug-encapsulated nanoliposomes: ERL- and DOX-encapsulated nanoliposomes (scale bar, 200 nm) (**a**), a single nanoliposome containing ERL and DOX-sulfate complexes (scale bar, 50 nm) (**b**), a high resolution- (HR-) TEM image representing DOX-sulfate crystals present inside the nanoliposome and ERL crystals in an outermost region of the nanoliposome (sale bar, 10 nm) (**c**), a SAED pattern of DOX-sulfate crystals and ERL crystals present in a nanoliposome (**d**), a single nanoliposome encapsulated with ERL (scale bar, 50 nm) (**e**), a HR-TEM image representing small crystals of ERL and amorphous domains in an outermost region of the nanoliposome (sale bar, 10 nm) (**f**), a SAED pattern of ERL crystals present in an outermost region of the nanoliposome (**g**), a single nanoliposome encapsulated with DOX (scale bar, 100 nm) (**h**), a HR-TEM image representing crystalline lattices of DOX-sulfate inside the nanoliposome (sale bar, 5 nm) (**i**), and a SAED pattern of DOX-sulfate crystals present inside the nanoliposome (**j**)

Figure 6 shows the morphology of the nanoliposomes observed by TEM. The samples for TEM observation were prepared using drop-casting of the ERL- and DOX-co-encapsulated nanoliposomes onto a carbon-coated copper grid and drying in air at RT. In order to compare the characteristics of the dual drug-encapsulated liposomes with the single drug-encapsulated liposomes, the morphology of the ERL-encapsulated or the DOX-encapsulated nanoliposomes was observed by TEM. As shown in Fig. 6a, the diameter of the dual drug-encapsulated nanoliposomes was less than 200 nm and the shape was nearly spherical. The liposome diameter observed through TEM analysis was coincident with the value measured using a particle size analyzer utilizing dynamic light scattering. Figure 6b shows the image of a single nanoliposome containing DOX-sulfate complexes inside the liposome. The complexes were assumed to be the crystals formed by attraction between protonated DOX cations and divalent sulfate anions [36]. Figure 6c shows a high resolution- (HR-) TEM image, which exhibited the drug crystals in the outermost region of the nanoliposome present in Fig. 6b. The image revealed that a highly crystalline lattice in the inner compartment of the nanoliposome and a less crystalline domain are having an amorphous domain close to the carbon on the grid [37]. The amorphous domain in the outermost region of the nanoliposome may be due to instability of the lipid bilayer upon exposure to the electron beam with high energy. Selected area electron diffraction (SAED) pattern present in Fig. 6d exhibited a regularity of bright diffraction spots, indicating that the crystals shown in Fig. 6c have a highly ordered crystalline lattice [38]. To compare the TEM analysis results of the dual drug-encapsulated nanoliposomes with those of the single drug-encapsulated nanoliposomes, the ERL-encapsulated nanoliposome was observed by TEM and the image is shown in Fig. 6e. The outermost region of the nanoliposome present in Fig. 6e was observed by HR-TEM, and the result is shown in Fig. 6f. From the result, it was found that the outermost region was composed of a number of small crystals and amorphous domains adjacent to the carbon on the grid. Figure 6g shows a SAED pattern of the crystals shown in Fig. 6f. The SAED pattern exhibited a ring made up of small spots arising from the individual crystals. Therefore, it is considered that the ERL intercalated between the lipid bilayer are present as small crystals with less ordered orientation [39]. On the other hand, as the other comparison, TEM analysis of the DOX-encapsulated nanoliposomes was carried out and the result is shown in Fig. 6h. The morphology and the diameter of the nanoliposome observed by TEM were similar to those of the dual drug-encapsulated nanoliposome. The HR-TEM image shown in Fig. 6i confirmed the crystalline lattice of the DOX-sulfate formed inside of the DOX-encapsulated liposome. The SAED pattern shown in Fig. 6j suggest that the crystals inside the liposome have a highly ordered crystalline lattice. These results verify that the diffractions of the ERL- and DOX-encapsulated nanoliposome by the electron beam were originated predominantly not by the ERL crystals in the lipid bilayer but by the DOX-sulfate crystals inside the nanoliposome. In summary, the morphology of the dual drug-encapsulated nanoliposomes and the crystals of each drug formed in the liposomes were identified by TEM, HR-TEM, and SAED analyses [40].

**Fig. 7:**
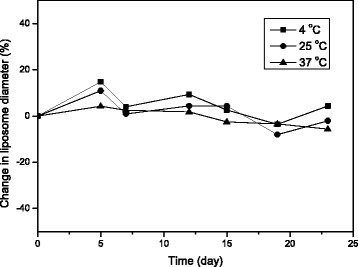
Stability of dual drug-encapsulated nanoliposomes incubated in PBS (pH 7.4) at different temperatures of 4, 25, and 37 °C, respectively: percent change in nanoliposome diameter relative to initial diameter as a function of incubation time

As a result of a physical stability study on the dual drug-encapsulated nanoliposomes, Fig. 7 shows the percent change in diameter of the nanoliposomes incubated in PBS (pH 7.4) at 4, 25, and 37 °C, respectively, as a function of the time. When incubated at 4 °C, in comparison with the initial diameter at day 0, the diameter of the nanoliposomes increased by 14.8% on day 5 and varied thereafter in a smaller extent of change in the diameter. The overall change of the diameter, however, was in a range of ± 15.0% during the stability test period. At 25 °C, the diameter of the nanoliposomes increased by 10.9% on day 5 and then decreased − 8.0% on day 19, indicating that the overall change was in a range of ± 10.0% during the test period. In addition, at 37 °C, the overall change in the diameter of the nanoliposomes was in a range of ± 6.0% during the test period, showing the smallest degree of change compared with the other test conditions. Based on the fact that in all three conditions tested, there was no significant change in the diameter of the nanoliposome, it is considered that the nanoliposomes prepared in our study have the physical stability in PBS (pH 7.4) for at least 3 weeks without aggregation or flocculation, which can affect therapeutic efficacy, targeting, and toxicity of the drug(s) encapsulated in the nanoliposomes. It has been acknowledged that the two important factors determining physical stability of nanoliposomes are *T*_*c*_ of phospholipid(s) and a content of cholesterol constituting the nanoliposomes [41]. The high stability of the nanoliposomes at 37 °C may be due to the low probability of phase transition of the phospholipid bilayer made up of DSPC, which has a high *T*_*c*_ and is utilized in general as a main lipid component in various liposome formulations. Also, a high composition ratio such as 40 wt% of cholesterol in the nanoliposomes seems to contribute to enhance the physical stability of the nanoliposomes [42]. On the other hand, nonetheless a low *T*_*c*_ (− 2 °C) of POPG having a double bond in its one acyl chain, a very low composition ratio such as 3 wt% of POPG had little effect on the stability of the nanoliposomes.

### Time-Differential Drug Release

**Fig. 8:**
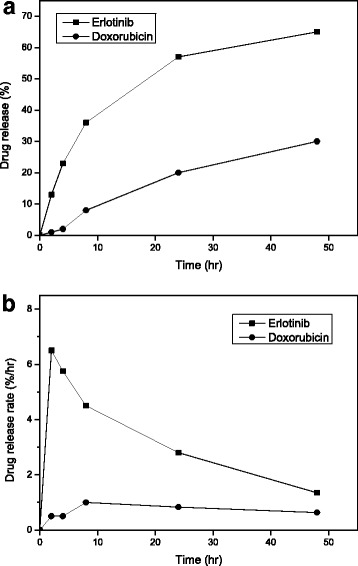
In vitro release profiles of ERL and DOX from dual drug-encapsulated nanoliposomes: time-differential release (**a**) and release rates of ERL and DOX (**b**) (*n* = 3, the data are presented as mean ± SEM)

There are a number of systems that can carry two kinds of drugs with different physicochemical properties on one vehicle. The representative examples are a micelle system containing the two drugs in the same compartment of the system, a polymer matrix system composed of different compartments containing each drug, and a liposome or a polymersome system containing each drug in the core and shell of the system, respectively [43–46]. In particular, when sequential actions of the two drugs are essential to cause a synergistic effect of the drugs, the time-differential release of each drug from the dual drug-encapsulated carrier is highly important. The in vitro time-differential release of DOX and ERL from the dual drug-encapsulated nanoliposomes was investigated, and the results are shown in Fig. 8. The releases of DOX and ERL from the nanoliposomes were monitored against a test medium, PBS (pH 7.4), under continuous stirring. The released amount of DOX or ERL after designated release test period was calculated measuring the fluorescence intensity of DOX and the absorbance of ERL with the fluorescence spectrometer and the UV-Vis spectrometer, respectively.

As shown in Fig. 8, the ERL release was 36 ± 0.01%, while the DOX release was less than 10% until 8 h of the release test period. In particular, during this period, the release rate of ERL was much faster than that of DOX. By 48 h, 65 ± 0.07% of ERL were released from the nanoliposomes in contrast to 30 ± 0.01% release of the DOX. The release rates of ERL and DOX are shown in Fig. 8b. Until 8 h, the release rate of ERL was more than 4% per hour in contrast to less than 1% per hour of the DOX release rate. After 8 h, the release rate of ERL was slowed down. Compared with the release of ERL, DOX showed a slow release and had an almost zero-order release rate. These results suggest that during the initial period of release test, much more amounts of ERL were released from the liposomes than those of DOX and there was a time-differential release between ERL and DOX. The sequential release of the drugs was thought to be originated from the difference between the physicochemical states of each drug in the liposomes [28, 32, 47]. These results on the dual drug-encapsulated nanoliposome system can contribute to translation of in vitro synergistic effects of two kinds of drugs into the clinic through overcoming both the difference between PK properties of each drug and the difficulty in targeting the same cancer cells in proper temporal sequence.

## Conclusions

As a dual drug delivery system, a nanoliposomal delivery system encapsulating both ERL and DOX was prepared and characterized. The liposome diameter was controllable by ultrasonication, and the sonication for diameter reduction was effective when carried out after the film-hydration and before DOX encapsulation. The nanoliposome diameter decreased remarkably during an initial period of ultrasonication. DOX was loaded into ERL-encapsulated nanoliposomes through pH- or AS-gradient method. AS-gradient method showed higher EE of DOX than pH-gradient, and the AS concentration for higher EE of DOX was determined. Equilibration of a mixture of DOX solution and ERL-encapsulated nanoliposomes in DOX-loading process was advantageous for EE increase of DOX. By HR-TEM and SAED analyses of the dual drug-encapsulated nanoliposomes, not only the highly oriented crystals formed between protonated DOX cations and divalent sulfate anions inside the liposome but also the less oriented small crystals of ERL in the outermost layer of the nanoliposome were identified. ERL and DOX co-encapsulated in the nanoliposomes showed a time-differential release, indicating much faster release of ERL than that of DOX from the liposomes. The elucidated preparation methods of the nanoliposomal dual drug delivery system can contribute to development of advanced dual drug delivery systems and translational researches of the combination therapies exhibiting synergistic effects via sequential actions of the drugs.

## Abbreviations

DOX: Doxorubicin
DSPC: 1,2-Distearoyl-*sn*-glycero-3-phosphocholine
EE: Encapsulation efficiency
EPR: Enhanced permeability and retention
ERL: Erlotinib
PK: Pharmacokinetic
POPG: 1-Palmitoyl-2-oleoyl-*sn*-glycero-3-phospho-(1′-*rac*-glycerol) (sodium salt)
SAED: Selected area electron diffraction
SEM: Standard error of mean
*T*_*c*_: Phase-transition temperature
TEM: Transmission electron microscopy
